# VCFtoTree: a user-friendly tool to construct locus-specific alignments and phylogenies from thousands of anthropologically relevant genome sequences

**DOI:** 10.1186/s12859-017-1844-0

**Published:** 2017-09-26

**Authors:** Duo Xu, Yousef Jaber, Pavlos Pavlidis, Omer Gokcumen

**Affiliations:** 1 Department of Biological Sciences, State University of New York at Buffalo, New York, 14260 USA; 2 Institute of Molecular Biology and biotechnology (IMBB), Foundation of Research and Technology--Hellas, Heraklion, Crete, Greece

**Keywords:** VCF, Phylogeny, FASTA, 1000Genomes, Anthropological genetics, Next generation sequencing data

## Abstract

**Background:**

Constructing alignments and phylogenies for a given locus from large genome sequencing studies with relevant outgroups allow novel evolutionary and anthropological insights. However, no user-friendly tool has been developed to integrate thousands of recently available and anthropologically relevant genome sequences to construct complete sequence alignments and phylogenies.

**Results:**

Here, we provide **VCFtoTree**, a user friendly tool with a graphical user interface that directly accesses online databases to download, parse and analyze genome variation data for regions of interest. Our pipeline combines popular sequence datasets and tree building algorithms with custom data parsing to generate accurate alignments and phylogenies using all the individuals from the 1000 Genomes Project, Neanderthal and Denisovan genomes, as well as reference genomes of Chimpanzee and Rhesus Macaque. It can also be applied to other phased human genomes, as well as genomes from other species. The output of our pipeline includes an alignment in FASTA format and a tree file in newick format.

**Conclusion:**

**VCFtoTree** fulfills the increasing demand for constructing alignments and phylogenies for a given loci from thousands of available genomes. Our software provides a user friendly interface for a wider audience without prerequisite knowledge in programming. **VCFtoTree** can be accessed from https://github.com/duoduoo/VCFtoTree_3.0.0.

**Electronic supplementary material:**

The online version of this article (10.1186/s12859-017-1844-0) contains supplementary material, which is available to authorized users.

## Background

The developments in next-generation sequencing technologies have now allowed us to study human genomic variation at the population scale. For example, 1000 Genomes Project alone sequenced more than 2500 individuals from diverse populations, uncovering more than 88 million variants including single nucleotide variants (SNVs), insertion-deletion variants (INDELs) (1–50 bp), and larger structural variants [1]. However, such large amounts of genomic data pose novel challenges to the community, especially for researchers working in fields where training for parsing and analyzing large datasets has not been traditionally established. One such field is anthropological genetics where the majority of studies have been locus-specific e.g., [2, 3], rather than genome-wide. One particular problem is to create manageable alignment files for loci of interest from whole genomic datasets to be compared to other sequences or outgroup species.

## Implementation

**Fig. 1:**
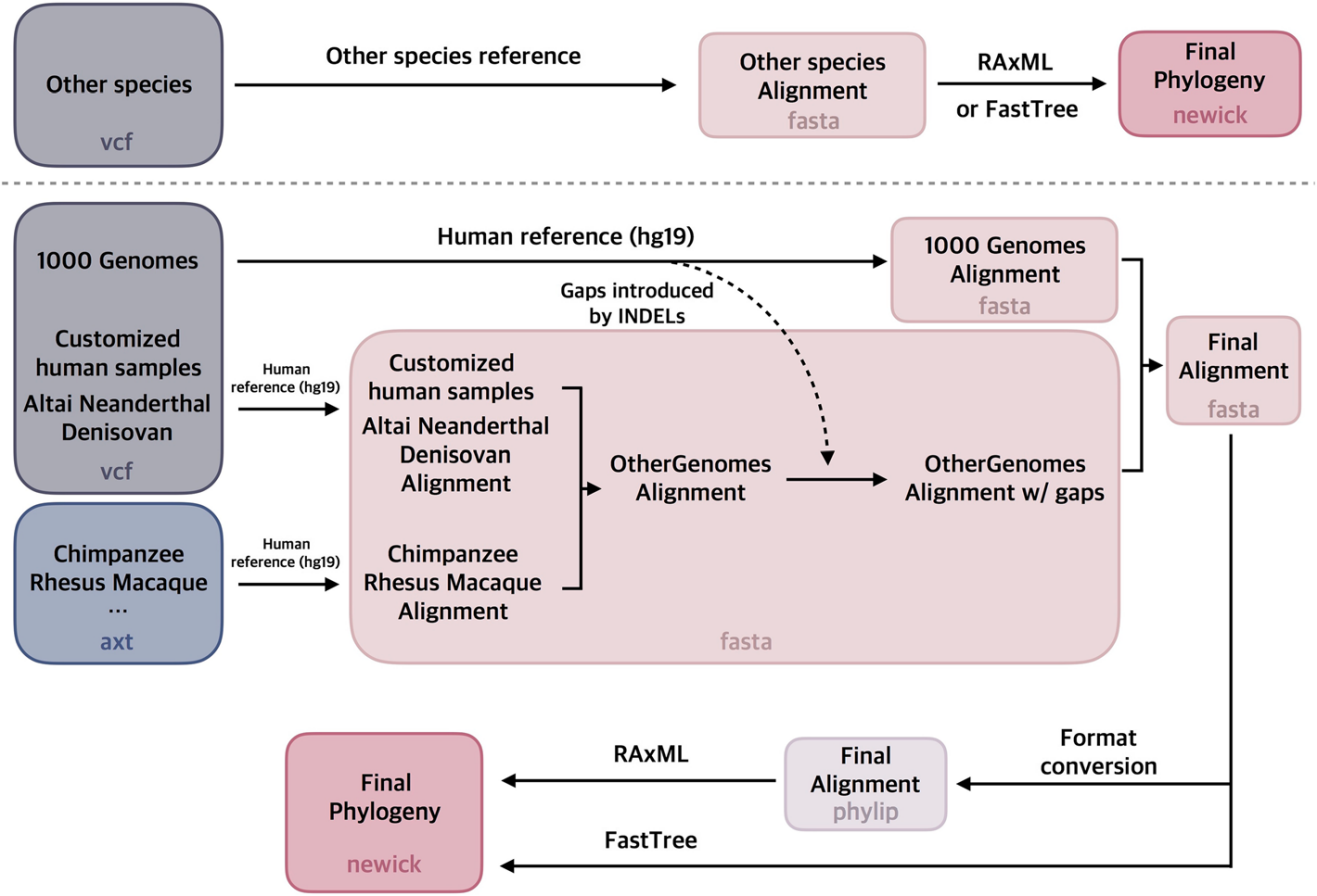
Workflow for VCFtoTree. Different colors stands for different file formats used in this study. The file formats are annotated on the bottom of each box. The upper panel shows the workflow for “others” when chosen from the main menu. The lower panel is the workflow for when “human” is chosen

**Fig. 2:**
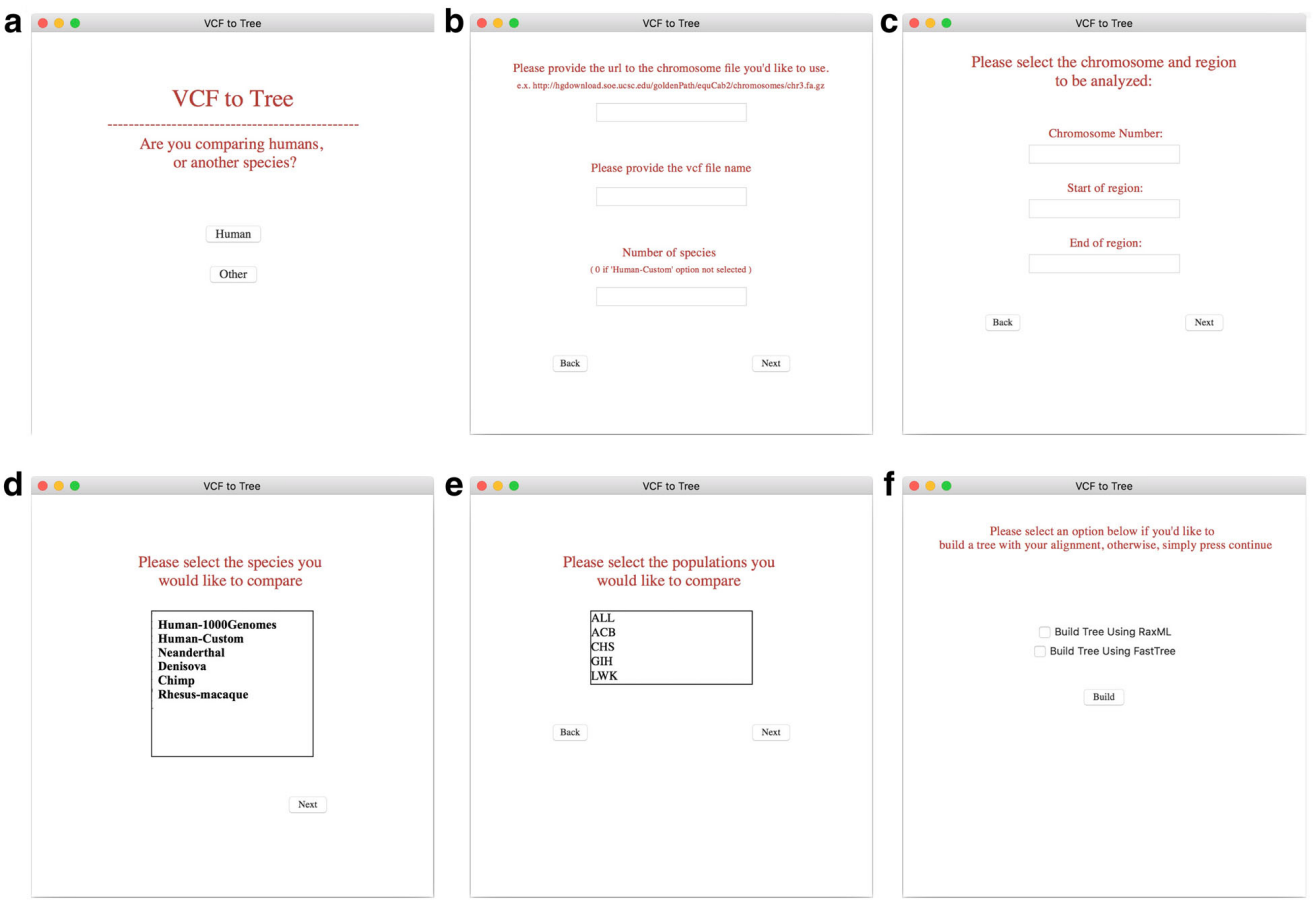
Graphic interface for VCFtoTree. From left to right, and from top to bottom are the interfaces of **VCFtoTree**. **a** Choose species that you want to study; **b** Provide the address (URL or local address) of your reference genome, vcf file, and enter the number of samples in your vcf file; **c** Enter your target region; **d** When you choose human in the main menu, you will be directed to this window to choose the dataset that you want to include in your alignment. “Human-1000Genomes” directly uses 1000 Genomes Phase 3 data, while you can use your own vcf file by choosing “Human-Custom”; **e** If you choose “Human-1000Genomes”, you will be directed to this window to choose the populations; **f**. Choose the phylogenetic tool you want to use for tree building. If neither were chosen, the program will only output the alignment

To address this need in the community, we present **VCFtoTree**, a user friendly tool that extracts variants from 5008 haplotypes available from 1000 Genomes Project, ancient genomes from Altai Neanderthal [4] and Denisovan [5], and generates aligned complete sequences for the region of interest (Fig. 1). Our pipeline also allows integration of sequences from reference genomes of Chimpanzee [6], and Rhesus macaque [7] to this alignment. Our program further uses these alignments to directly construct phylogenies. We constructed a graphical user interface so that our pipeline is accessible to a broader user community where users can choose species and populations of interests, or load their custom files (Fig. 2). For more experienced researchers, we provide all the scripts used in the program on https://github.com/duoduoo/VCFtoTree_3.0.0. Those scripts can be easily modified to add other species or populations. The resulting alignments from our pipeline can also be integrated into other applications that require alignments, such as calculation of population genetics summary statistics or genome-wide applications, such as phylogenetic analyses of windows across the entire chromosomes.

### Data sources & aligning sequences to human reference genome (hg19)

The modern human variants used in **VCFtoTree** are from 1000 Genomes Phase 3 final release. This dataset contains single nucleotide, INDEL, and structural variants (SVs) from 2504 individuals from 26 worldwide populations [1]. Please note that for annotations, we followed exactly the nomenclature that is used in 1000 Genomes Project. For example, INDELs are defined as insertions and deletions that are smaller than 50 bp. Larger variants were categorized as SVs. The variation calls (i.e., their location on the hg19 reference assembly and the non-reference alleles) are available in Variant Call Format (VCF) in a phased manner [8]. Our program fetches and indexes these VCF files for a specific region of interest designated by the user. For this, we integrated tabix from SAMtools [9] to our pipeline. We use a similar strategy to fetch and parse single nucleotide variants from ancient hominin genomic variants that are available from two high-coverage genomes, Altai Neanderthal (http://cdna.eva.mpg.de/neandertal/altai/AltaiNeandertal/VCF/) [4] and Denisovan (http://cdna.eva.mpg.de/neandertal/altai/Denisovan/) [5]. These variant calls are available also in Variant Call Format through the Department of Evolutionary Genetics of Max Planck Institute. It is important to note that our pipeline does not integrate the INDELs in these ancient genomes to the final alignment and phylogeny building. Instead, we report the INDELs in the specified region in two files: “Indels_Altai.txt” and “Indels_Denisova.txt”.

Chimpanzee and Rhesus Macaque are often used as outgroups in human evolutionary genetics studies [10]. Thus, our program integrates sequences from Chimpanzee and Rhesus Macaque reference genomes to our alignment files. Specifically, we use the pairwise alignments for Human/Chimpanzee (hg19/panTro4) [6] and Human/Rhesus (hg19/rheMac3) [7] directly from the UCSC genome browser [11]. Since our goal is to delineate genetic variation in humans, we only keep the alignment gaps that have been identified in human sequences, even though this information might be missing in Chimpanzee and/or Rhesus sequences. In other words, we are using human reference genome (hg19) as the reference for our final alignment with regards to incorporating nonhuman species. It is important to note that this approach may underestimate the divergence between humans and nonhuman primate sequences in cases where there is human-specific deletions in the region of interest.

### Transforming the variant calls to complete sequences.

Once our program fetches and sorts all the variant calls from designated sources as described above, our pipeline transforms these variant calls to complete sequences for alignment. There are computational tools to manipulate VCF files from 1000 Genomes Project (e.g., vcf-consensus in vcftools [8], “vcf2diploid” function in GATK [12]). However, these tools are not able to construct alignment of all 5008 haplotypes available in 1000 Genomes Project dataset for a given locus. A such, we devised the python script vcf2fasta.py in **VCFtoTree** to transform the variant calls into complete, aligned sequences as we describe below.

**Fig. 3:**
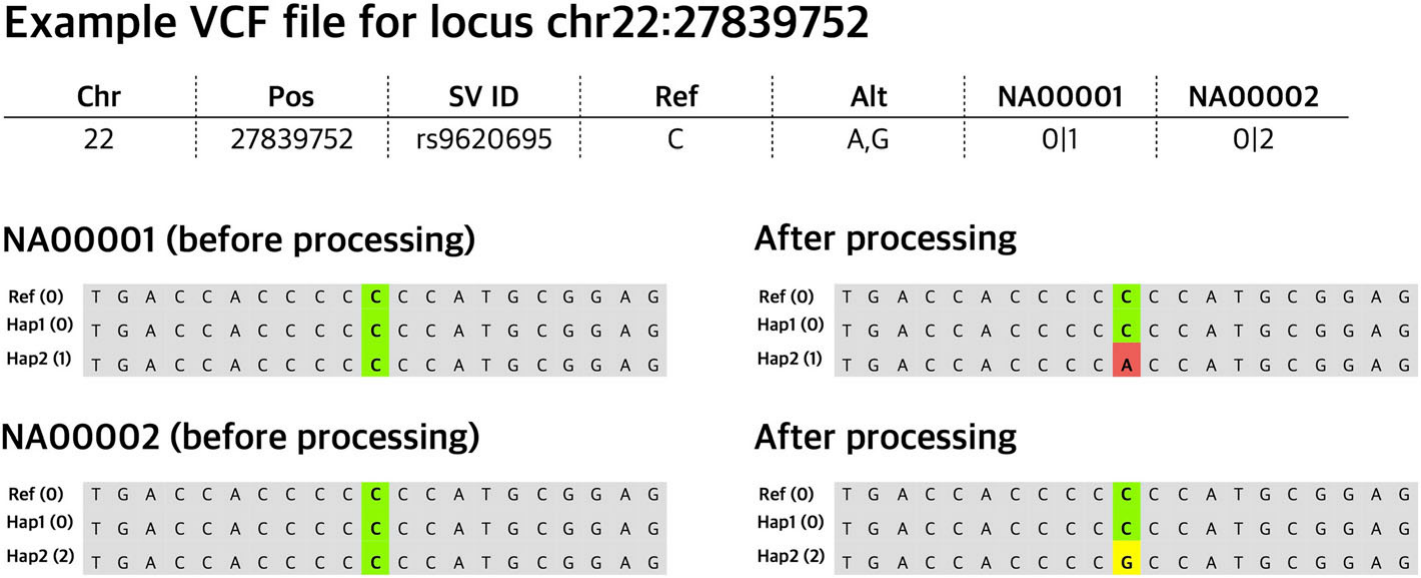
Scheme for transforming 1000 Genomes Project’s variations to sequence for each individuals

1000 Genomes dataset is phased. As such, for each individual genome there are two haplotypes. For each variable loci in each haplotype, there is a designation in the VCF file where 0 stands for the reference allele, while 1, 2, 3, 4 stand for the first, second, third, and fourth alternative alleles, respectively. Our pipeline extracts this information for a user-designated region in the genome. Then it regenerates the sequences of the individual haplotypes by changing the reference genome sequence in this region. Most variations have only two alleles. However, to explain how our pipeline deals with a more complicated, and not uncommon situation, we provide an example. Let’s say, at a particular locus where the reference allele is “a”, there are two alternative alleles “C” and “T”. For an individual sample, the VCF file designates the allele in a given chromosome as 0, 1, or 2, corresponding to the reference allele “a”, “C”, and “T”, respectively. Therefore, when a genotype is designated as 0|2 for this locus, the first haplotype of this sample carries the reference allele (“a”), while the second haplotype carries a third allele (“T”). Based on this information, our script generates two sequences based on the reference genome to represent these individual haplotypes. For the first haplotype, the script leaves that position as it is (“a”), but for the second haplotype, the script replaces “a” with a “T” to represent the variation in this haplotype (Fig. 3). This will be done for all the haplotypes and for all the single nucleotide variants within the designated region.

Our method of transforming VCF files to complete sequences applies to the Neanderthal and Denisovan genomes as well. However, these two archaic hominin genomes are not phased. To address this issue and to ensure that we capture variants that truly differ from the reference genome, we only considered homozygous variants from these genomes. Given that these ancient genomes are extremely homozygous due to recent inbreeding [4, 5] the impact of this bias is minimal. In other words, there are very few (if any) regions reported in the Neanderthal or Denisovan genomes that show heterozygosity of a derived variant shared with modern humans [4, 13]. However, it is still a possibility that in a small number of regions, our pipeline may underestimate the divergence between modern and these ancient hominins, or miss signals of heterozygosity in Neanderthal and Denisovan genomes.

### Incorporating short INDELs and structural variants

**Fig. 4:**
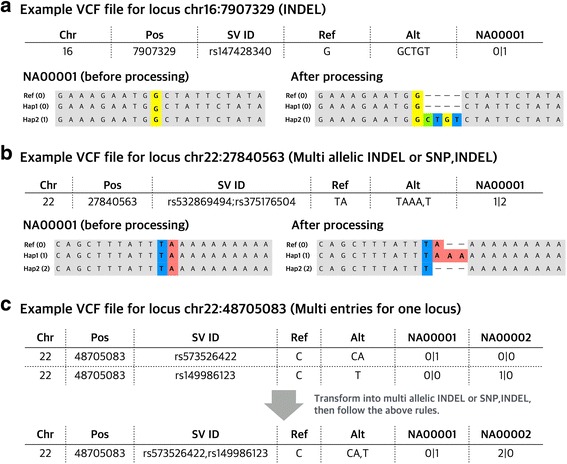
Scheme for transforming INDELs and complex variant types from 1000 Genomes Project into sequence for each individual. **a** Example showing how **VCFtoTree** transforms INDEL variant; **b** Example showing how **VCFtoTree** transforms multi allelic INDEL or variant type SNP,INDEL; **c** Example showing how **VCFtoTree** transforms multiple variants on the same locus. It transform the VCF line into a multi allelic VCF line, then follow the rule for multi allelic variant

Besides the single nucleotide variants, there are other variant types involving more than 1 base pairs, including INDELs and genomic structural variants. In such cases, simply adding those multi-base pair alternative alleles to the reference genome haplotype would cause frameshift in the alignments. Realigning these sequences is computationally inefficient and often introduces errors. To address this issue, first, we considered short INDELs, which are <50 bp sequences that are missing or inserted in a given haplotype annotated as “VT = INDEL” in 1000 Genomes VCF files. Briefly, our pipeline adds the insertions to the reference sequence according to their positions indicated by the VCF file to generate the sequences for these haplotypes. This essentially increases the sequence length of our overall alignment file. For the haplotypes that do have this insertion sequence, we filled the space by adding “-” to the corresponding sites. For the haplotypes with deletions that are smaller than 50 bp, we simply indicated the deleted sequence with replacing the deleted sequences with “-” in the reference haplotype (Fig. 4a).

The phase 3 dataset of 1000 Genomes detected more than 60,000 structural variants which include large duplications, deletions, copy number variations, inversions, mobile element insertions, etc. The breakpoints of structural variants (unlike INDELs) vary and often not definitive. Moreover, the insertion sites of most duplications and mobile element insertions are not known. As such, our current pipeline is not equipped to reliably integrate structural variants in the alignment files. We ignore the structural variants for constructing our alignment. However, for the researchers to be able to assess the variation in the region fully, we provide the structural variants in the user-designated regions into the log file (log.txt). It is important to note that structural variants often resides in “complex”, repeat-rich regions of the genome where there are other alignment issues [14]. It is a general challenge in the field and currently we recommend to manually check the alignments in such regions where structural variants are reported.

### Incorporating complex variations

In 1000 Genomes VCF files, there are several loci where variant calls are complex: i.e., they harbor more than one kind of variant type or overlapping INDELs, or multiple entries were made for the same locus. Here, we list the approaches that we took to integrate these complex variants in our pipeline:

- Locus with multiple variant types: There are some loci that can have an INDEL and a single nucleotide variant for different haplotypes. These sites are designated in the VCF file as “VT = SNP,INDEL”. In such cases, we convert the single nucleotide variant call to an INDEL format and treat this particular VCF line as a multiallelic INDEL as described above and as exemplified in Fig. 4b.

b) Locus with multiple entries: There are some cases where 1000 Genomes VCF files report different alleles affecting the same locus in different lines, rather than designating them in a single line as multiallelic variants. In these cases, our pipeline combines these variants, creating a multiallelic variant line and then treats them as such (Fig. 4c).

c) Complex regions with overlapping INDELs: Some highly repetitive sequences may vary in the number of repeats and they are designated as overlapping INDELs. We were able to integrate a subset of those where the multiple haplotypes are missing different sizes of sequences that are present in the reference genome (overlapping deletion INDELs). Briefly, our pipeline combines these events as multiallelic INDEL variants and then treats them as such. However, our pipeline cannot handle overlapping INDELs with sequences that are not present in the reference genome. If the region specified harbors such novel insertions overlapping with other INDELs, our program will not run and instead return an error message. The overall impact of this shortcoming is small given that there are only 892 distinct cases of such overlapping insertion INDELs reported in 1000 Genomes Project, excluding X/Y chromosomes Additional file 1: Table S1), most of which are in the centromeric or telomeric regions of the genome.

### Integrating sequences from 1000 genomes, archaic hominins, chimpanzee and rhesus into final alignment files

After generating the alignment for 5008 haplotypes from the 1000 Genomes Project, our pipeline can add variation data from Altai Neanderthal and Denisovan genomes, as well as Chimpanzee and Rhesus Macaque reference sequences to the alignment. Since, the archaic hominin variant calls were directly made from human reference genome, the alignment is automatic. That is we treat the VCF files from these ancient genomes similar to the 1000 Genomes VCF files, with the exception that we only consider homozygous variants as described above. For the nonhuman primates, we use existing pairwise alignment files for chimpanzee and rhesus macaque reference genomes to human reference genomes for a given region to construct the alignments. The challenge here is to incorporate all length changes that we introduced to the alignments while we integrate insertion INDELs. To do this, we use a custom python script in our pipeline to add these additional sequences to the archaic hominin and nonhuman primate genomes as gaps (“-”) before integrating these sequences to our alignment.

### Integrating custom vcf files and reference genomes from nonhuman species

Even though we primarily intend our application to be used for human genomes, we also implemented two options to broaden its scope. First, we allow researchers to load their own vcf file for phased genomes. Second, for nonhuman species, the researchers can also load any given reference genome to the program to work along with vcf files from that species. In the first options screen, it is possible to choose “other” and in the next screen locations of the reference input file (.fa.gz) and the desired vcf file (.vcf.gz) can be designated. Other than the input reference and variation files, all the algorithms, corrections and exceptions that we outlined above remain the same. There are three considerations that need to be noted here. First, most nonhuman reference genomes are not very high quality and may cause problems given that our analysis pipeline depends on the accuracy of the reference genome for constructing alignment output. We have not tested our approach extensively with nonhuman reference genomes. Second, we assume that the variation annotations in the custom vcf files will be identical to those used in 1000 Genomes Project. Third, it is important to remind that our approach only works with phased genomes. Even though there are not many phased nonhuman genomes currently available, we foresee that in the near future they will be. Our tool will be ideal to analyze such data.

### Constructing phylogeny using RAxML and FastTree

#### Alignment outputs

The initial alignment constructed is in FASTA format. Our pipeline also uses a python script to transform the format of FASTA to Phylip format. We provide both alignment formats as output files.

#### Constructing phylogeny

The last step for our pipeline is to build the phylogeny is to run RAxML [15] or FastTree [16]. In this step, by default the RAxML is performed under GTR + GAMMA model on 2 cores of a personal computer, and the FastTree is compiled without the limit on branch length precision, and performed under GTR + GAMMA model. However, the parameters can be easily modified in the script for your own purpose. After the phylogeny constructing process concludes, our pipeline will output the final phylogeny (“bestTree”) with filename extension “.newick”. To conveniently visualize this large phylogeny file, we recommend Dendroscope [17] or Archaeopteryx [18], which are two user friendly tools for viewing large phylogenies.

## Results and discussion

**Fig. 5:**
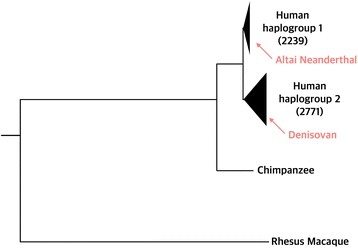
Phylogeny of ERAP2 generated by VCFtoTree. The tree was rooted by midpoint rooting, and visualized by Archaeopteryx [18]. The phylogeny is for 5008 human haplotypes, Altai Neanderthal and Denisovan variations, as well as chimpanzee and rhesus macaque reference genomes. The size of the black triangles are proportional to the number of haplotypes in that lineage. The phylogenetic locations of Neanderthal and Denisovan genomes were highlighted by red arrows

**VCFtoTree** emerges from our own needs in our laboratory and we used previous versions of this pipeline in our recent publications [19–21]. We also applied our pipeline to gene regions with signatures of balancing selection, EDAR [22], ERAP2 [23], NE1 [24]. As expected, the phylogenies created by our pipeline clearly showed two divergent, separated lineages for such regions, which is a hallmark of balancing selection [25] (Fig. 5, Additional file 2: Fig. S1).

**VCFtoTree** is comparable to other phylogenetic analyses tools, such as Network [26] or Arlequin [27]. The improvement we provide is to handle large amount of whole genome sequencing data from thousands of individuals, and also other species. For 1000 Genomes Project data, we were able to skip the tedious input data preparation step. Instead, the data will be automatically downloaded. The only input needed from users is the species and populations of interest, as well as the target genomic region. **VCFtoTree** can also take phased customized VCF files from human and other species which makes it a more flexible tool.

The run time of **VCFtoTree** depends primarily on the bandwidth available to access 1000 Genomes variation and ancient genome datasets. It is important to note that ancient genome sources do not provide an index and hence the entire ancient chromosome data are downloaded, which slows down the process more than downloading from indexed 1000 Genomes dataset. The parsing of the data with our custom code to construct alignments is relatively fast. To give you an example, it takes less than 5 mins (0:04:34) to output an alignment file for a 10,000 bp region for 2504 individuals running on a ISO system with 2.6 GHz Intel Core i5 processor and 8 GB 1600 MHz DDR3 memory. The second major bottleneck in timing is the tree building step. Especially RAxML requires rather long run times for large regions. The specifications and their run-time specifications can be found in Stamatakis 2006 [28]. FastTree-based phylogenies are much faster to run and can largely decrease the run time for tree building step.

## Conclusion

Next-generation sequencing platforms increased the amount of genomic data tremendously. The critical bottleneck in anthropological genetics research has consequently shifted from production of data to analyses of data. For now, most of the available computational tools (e.g., vcftools [8]; GATK [12], etc.) are used to parse large datasets for further custom-designed computational pipelines. As such, starting from whole genome sequencing variant calls to a phylogenetic analysis of a given locus in humans requires a certain level of programming knowledge. Recently emerging tools such as UCSC Genome browser [11], Geography of Genetic Variant Browser(http://popgen.uchicago.edu/ggv/), Galaxy [29] and 1000 Genomes Selection Browser [30], among others are very helpful for non-computational users to study single loci. **VCFtoTree** complements such tools by providing a graphic user interface to investigate the haplotype structure of a locus at the population level while generating alignments for further analyses in software such as MEGA [31] and DNAsp [32].

In addition to within species analysis, **VCFtoTree** can also be useful in cross-species analysis. By using **VCFtoTree**, users can resolve the haplotype structure for the given region, finding the haplotype groups that compose the population. Then by choosing 1–2 representative haplotypes from each haplogroup, users can use commonly used multiple sequence alignment tools such as MEGA [33], Seaview [34], to realign sequences for cross-species comparison. This method has been successfully applied to evolution studies for *MUC7* [21], *FLG* [19] and *LCE3BC* [20]. We continuously work on new ways to analyze emerging large datasets and we hope to implement those new insights and datasets to **VCFtoTree** as they become available. Overall, we believe that our pipeline will be useful for researchers in anthropological and evolutionary genomics, who are interested in locus-specific analyses.

## Availability and requirements

**Project name:** VCFtoTree.

**Project home page:** https://github.com/duoduoo/VCFtoTree_3.0.0.

**Operating system:** Mac OS El Capitan V10.11.5 or later.

**Programs required:** samtools, tabix, wget.

**Programming languages:** Python, Unix.

**License:** Not applicable.

## Additional files

Additional file 1: Table S1. Complex regions with multiple overlapped INDELs. (TXT 25 kb)

Additional file 2: Figure S1. Phylogenies generated by VCFtoTree. a) EDAR [22]; b) NE1 [24]. (JPEG 231 kb)

## Abbreviations

INDELs: Insertion-deletion variants
SNVs: Single nucleotide variants
SVs: Structural variants
VCF: Variant call format
VT: Variant type
