# Supplementary figures and images for: VCFtoTree: a user-friendly tool to construct locus-specific alignments and phylogenies from thousands of anthropologically relevant genome sequences
Source: BMC Bioinformatics. 2017 Sep 26;18:426. doi: 10.1186/s12859-017-1844-0 (PMC5615795; doi:10.1186/s12859-017-1844-0)

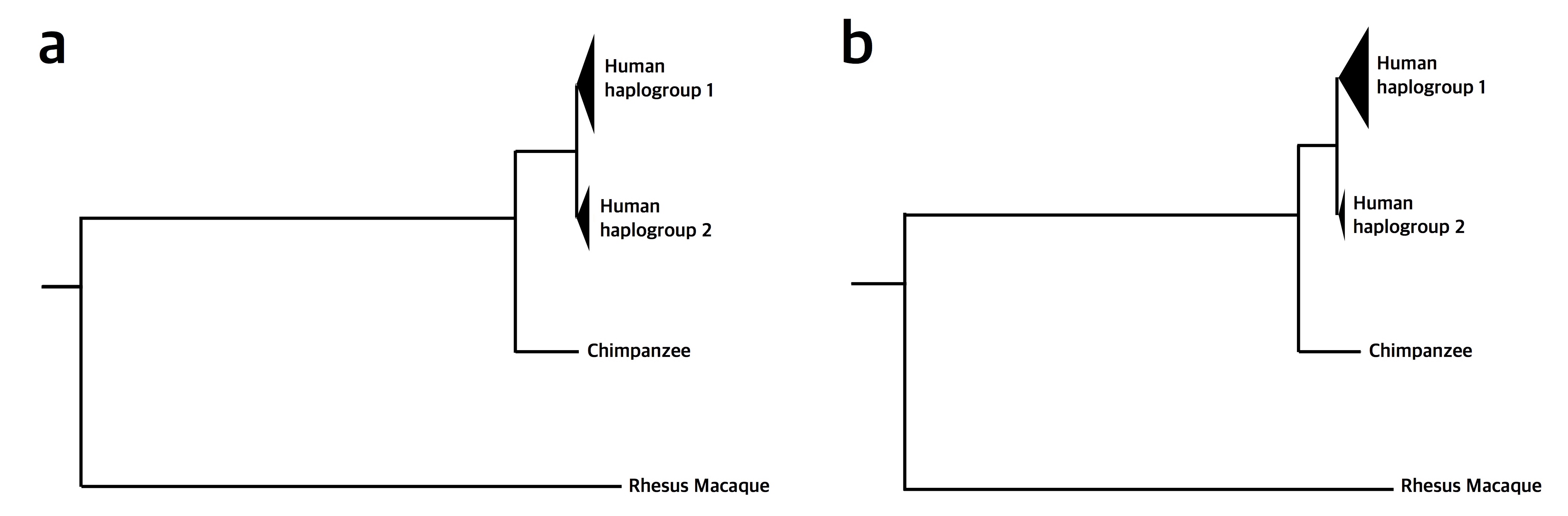

Supplement: Supplementary file 2 — Phylogenies generated by VCFtoTree. a) EDAR [22]; b) NE1 [24]. (JPEG 231 kb) [file 12859_2017_1844_MOESM2_ESM.jpg]
